# Equine pericardial roll graft replacement of infected pseudoaneurysm of the ascending aorta

**DOI:** 10.1186/1749-8090-7-54

**Published:** 2012-06-14

**Authors:** Hiroshi Kubota, Hidehito Endo, Mio Noma, Hiroshi Tsuchiya, Akihiro Yoshimoto, Yu Takahashi, Yusuke Inaba, Mitsuru Matsukura, Kenichi Sudo

**Affiliations:** 1 Department of Cardiovascular Surgery, Kyorin University, Tokyo, Japan

**Keywords:** Equine pericardium, Biomaterial, Infection, Aortic aneurysm, Surgery, Pseudoaneurysm

## Abstract

The standard procedure for treating infected aortic aneurysms is to resect the infected aorta, debridement of the surrounding tissue, in situ graft replacement, and omentopexy. However, the question of which graft material is optimal is still a matter of controversy. We recently treated a patient with an infected ascending aortic aneurysm. Because of previous abdominal surgery, the omentum was unavailable. The ascending aorta was replaced in situ with equine pericardial roll grafts. The patient is alive and well 29 months after the operation.

## Background

How should we treat the infected thoracic aorta surgically? Usually, after complete resection of the infected aorta and debridement of the surrounding tissue, in situ graft replacement and omentopexy is performed. As a graft material, Dacron grafts, rifampicin-soaked Dacron grafts, cryopreserved arterial homografts are accepted clinically. Omentopexy is thought to be a most reliable barrier to prevent the recurrence of graft infection because unguarded foreign materials are easily propagated by bacteria. When the omentum is unavailable because of previous abdominal surgery, how should we treat the infected aorta? We experienced a case with a infectious pseudoaneurysm at the ascending aorta due to postoperative septicemia and the omentum was unavailable because of previous gastrectomy to treat gastric cancer. The aorta was replaced in situ with equine pericardial roll grafts without omentopexy.

## Case presentation

**Figure 1:**
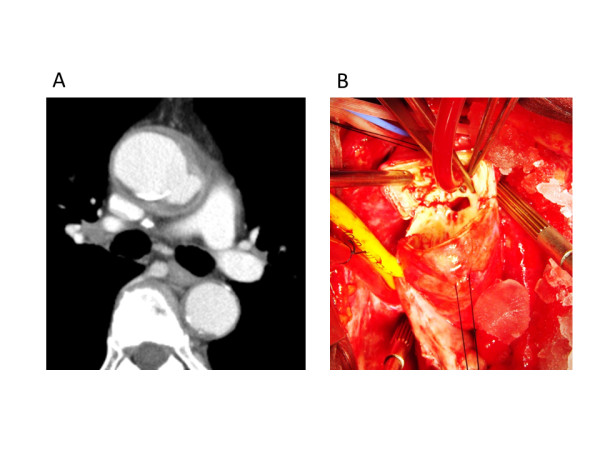
** A. Preoperative computed tomography scan (case 1).** A pseudoaneurysm and abscess were detected in the ascending aorta. **B**. Intraoperative view (case 1). The ascending aorta was “punched out”. The abscess extended just above the main trunk of the left coronary artery.

Case: A 77-year-old male came to our hospital because of a fever and chest pain. The patient had one month previously undergone subtotal gastrectomy and omentectomy to treat early gastric cancer. His leukocyte count was 10,500/μl, and his serum C-reactive protein level was elevated to 21.6 mg/dl. A blood culture was positive for methicillin-sensitive *Staphyrococcus aureus* (MSSA). Computed tomography of the chest revealed a rapidly growing pseudoaneurysm and abscess formation around the pseudoaneurysm (Figure 1-A). Systemic examination revealed no obvious primary focus of the infection; teeth, urinary, or respiratory system. Previous abdominal operation, and central venous and urinary catheter insertion were possible to be the origin of the infection. Urgent ascending aortic replacement without omentopexy was performed.

### Operative procedure

**Figure 2:**
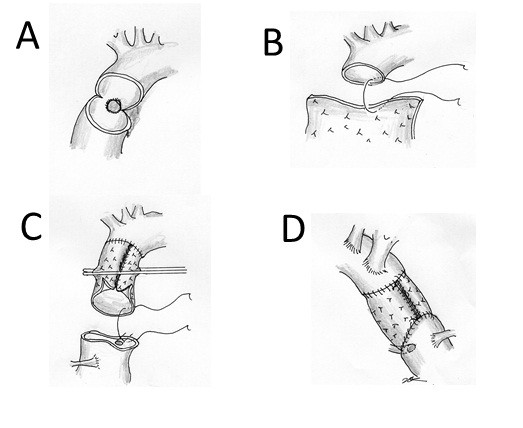
** Operative procedure.A**, **B**. The ascending aorta was transected immediately proximal to the orifice of the innominate artery. The diameter of the ascending aorta was 30 mm. A 10 cm × 10 cm sheet of equine pericardiaum was prepared, and after suturing it to the posterior side of the transected aorta, it was rolled up by continuous suturing from posterior to anterior. **C**. When the corners of the pericardium met, the suture was tied, and the same thread was used to stitch the two sides of the pericardium continuously to form a cylinder. **D**. The proximal ascending aorta was transected obliquely, and after trimming the pericardial sheet, a beveled proximal anastomosis was created.

**Figure 3:**
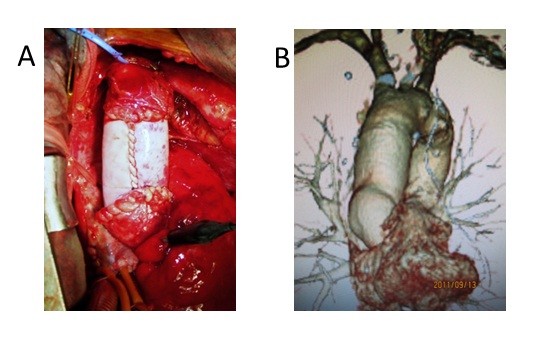
** Intraoperative photo and postoperative computed tomography.A**. The pseudoaneurysm and abscess were resected and debrided. The infected ascending aorta was replaced by the equine pericardial roll graft without omentopexy. **B**. The postoperative computed tomography 24 months after the operation showed no dilatation of the graft.

On July 16, 2009, the pericardium was opened through a median sternotomy. There was a large purulent pericardial effusion. Cardiopulmonary bypass was established with cannulations via the right femoral artery, superior vena cava and inferior vena cava. When the patient’s tympanic membrane temperature had fallen to 20 degrees centigrade, the ascending aorta was opened with the patient in circulatory arrest, and it was transected just immediately proximal to the orifice of the innominate artery (Figures 1-B, 2-A). The aortic segment containing the aneurysm was removed. A 10 cm × 10 cm equine pericardial sheet (XGA-400; Edwards Lifesciences, Irvine, CA, USA) was sutured to the posterior side of the transected aorta and rolled up by continuous suturing from posterior to anterior with 4-0 polypropylene (Figure 1-B). When the corners of the pericardial sheet met, the suture was tied, and the same thread was used to stitch the two sides of the pericardial sheet continuously to form a cylinder (Figure 2-C). The graft was clamped, the cardiopulmonary bypass was resumed, and the patient was warmed. The abscess and surrounding tissue were debrided. Because the abscess cavity behind the posterior side of the ascending aorta extended to just above the main trunk of the left coronary artery, the proximal ascending aorta was transected obliquely, the pericardial sheet was trimmed, and a beveled anastomosis was created by using a 4-0 polypropylene continuous suture (Figure 2-D, Figure 3-A). The aortic cross clamp time was 62 minutes and the circulatory arrest time was 40 minutes.

Because the diameter of the transected aorta was about 3 cm, 10 cm × 10 cm pericardial sheets were used as is. An each margin to sew up was calculated as (10 - 3 π) / 2 = 0.3 cm. Biological glue containing a rifampicin was sprayed on the grafts and anastomoses. No foreign materials were used to reinforce the anastomoses.

## Consent

Written informed consent was obtained from the patient for publication of this report and any accompanying images.

## Results

**Figure 4:**
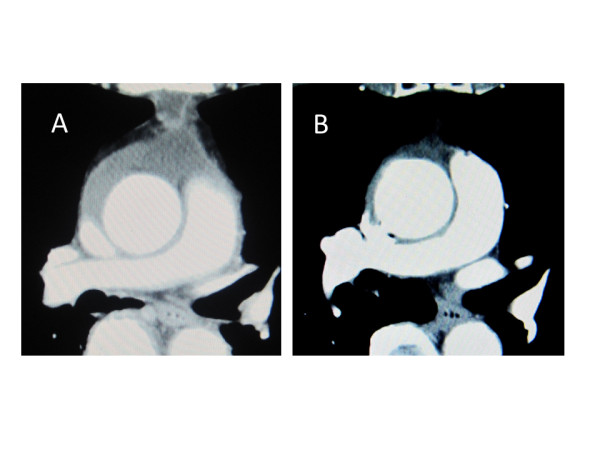
** Postoperative computed tomography.A**. Two weeks after the operation, perigraft fluid collection was detected. **B**. Two years after the operation, perigraft fluid collection was disappeared.

Both the cultures of the resected aorta and pericardial effusion were positive for MSSA. The postoperative course was uneventful. The patient recovered from surgery well and had no neurological deficits. Postoperatively, 1.0 g/day of vancomycin hydrochloride and 1800 mg/day of clindamycin hydrochloride were administered intravenously for 17 days. The patient’s leukocyte count and C- reactive protein level gradually decreased and reached to the normal range at 14th postoperative day. Because of a drug eruption and normalized laboratory inflammatory data, they were stopped and no antibiotic treatment was continued. Although, postoperative computed tomography two weeks after the operation showed perigraft fluid collection, it disappeared 24 months after the operation (Figure 4-A, B). As of 29 months after surgery the patient are alive and well, and has been no recurrence of the infection. Three-dimensional computed tomography which was taken 24 months after the operation demonstrated well functioning roll grafts and absence of stenosis, dilatation, or thrombus formation (Figure 3-B). No anticoagulants or antiplatelet drugs were administered.

## Discussion

Resection of the infected aorta, debridement of the surrounding tissue, graft replacement, and omentopexy is generally considered the standard treatment to cure infected aortic aneurysm. However, the most suitable graft materials to be used to replace the infected aorta are a matter of controversy. Dacron grafts, rifampicin-soaked Dacron grafts are accepted clinically [1-3]). Although cryopreserved arterial homografts are excellent material to treat infected aortas, but the supply in Japan is inadequate, and it is difficult to be in time for urgent operation. The autogeneous pericardium has been reported to treat infective endocarditis, but the surface area is not enough to reconstruct the great vessels [4]). Further, considering the importance of closing the pericardium to avoid the recurrence of the infection, it is better to preserve the autogeneous pericardium. Instead of these materials, an equine pericardium was used to reconstruct the ascending aorta based on the case reports described by Yamamoto et al [5]). They used the equine pericardium in the locally-infected field when repairing infected abdominal aortic aneurysm rupture and have confirmed excellent durability of the graft without graft infection in the long-term follow-up. They also reported a case of successful in situ replacement of the thoracic descending aorta with an equine pericardial roll graft with left lower lung resection for an aortobronchial fistula due to aortic rupture caused by the infection due to α–streptococcus [6]). Omentopexy was not performed in the patient, because omental mobilization was considered impossible due to a past history of laparotomy for an esophageal hiatal hernia.

As for xenopericardium, the feasibility of crimped bovine pericardial conduit to reconstract the aorta was reported [7]). Czerny et al. reported excellent result of the bovine pericardial tube graft to treat prosthetic graft or endovascular graft infection in 15 patients. They mention that xenopericardial tube graft may be superior to cryopreserved homografts because the likelihood of calcification seems to be less important and another advantage of customized xenopericardial tissue is the availability, which turns out to be problematic with homografts [8]). A pericardial sheet is soft and easy to handle. It could be made to be cylindrical intraoperatively by rolling it up and gave us a good operative field. The graft dilatation, mural thrombus formation, and recurrence of the infection are concerns during long-term follow up. Enhanced computed tomography may be the most suitable examination for follow-up. The need for treatment with anticoagulant or antiplatelet agents is also a matter of controversy. We propose that, as we reported previously, patients who have undergone surgical reconstruction of the arch vessels, be treated with an anticoagulant or antiplatelet drug to prevent strokes and graft stenosis due to mural thrombi [9]. The type of pathogen as well as the graft material affects the prognosis. Yamamoto et al. alerted that the inner layer of the equine pericardial roll graft in a patient who underwent in-situ replacement of an infected ruptured abdominal aorta was colonized and damaged by methicillin-resistant *Staphylococcus aureus* (MRSA) [10]). They suggested that, under MRSA sepsis, the equine pericardium might not have an enough barrier against bacterial colonization, resulting in a possibility of structural instability due to tissue destruction.

## Conclusion

In conclusion, the equine pericardial roll graft replacement without omentopexy to treat the infected aortic pseudoaneurysms is a simple procedure. By accumulating clinical cases, when its long-term durability will be confirmed, it may demonstrate the advantages of this material as one of the choices of treatment for infected aortic aneurysms.

## Competing interest

The authors declare that they have no competing interests.

## Authors’ contributions

HK, HE, MN, HT, AY conceives of the study, and participated in its design and coordination. YT, YI, MM and SK participated in the sequence alignment. All authors read and approved the final manuscript.
